# Protective Effects of Mycotoxin Biodegradation Agents on Pigeon Health and Gut Microbiota

**DOI:** 10.3390/toxins18070299

**Published:** 2026-07-10

**Authors:** Hanke Zhao, Changfeng Xiao, Hui Jiang, Lihui Zhu, Junhua Yang, Fang Gan

**Affiliations:** 1 Institute for Agro-Food Standards and Testing Technology, Shanghai Academy of Agricultural Sciences, Shanghai 201403, China; zhaohanke@saas.sh.cn; 2 Institute of Animal Husbandry Veterimary Science, Shanghai Academy of Agricultural Sciences, Shanghai 201403, China; xiaochangfeng@saas.sh.cn (C.X.); jianghui@saas.sh.cn (H.J.); 3 College of Veterinary Medicine, Nanjing Agricultural University, Nanjing 210014, China; ganfang@njau.edu.cn

**Keywords:** mycotoxins, mycotoxins adsorbent agent, liver inflammation, gut microbiota

## Abstract

Mycotoxin contamination in feed poses a major threat to animal health and productivity, particularly in pigeons. This study evaluated the protective effects of three mycotoxin biodegradation agents (MBAs) in pigeons subchronically co-exposed to a mycotoxin-contaminated (MYC) diet for 60 days. The MYC diet was prepared by mixing 3% naturally mold-contaminated corn with 97% basal diet, yielding final dietary concentrations of fumonisin B1 (7829.41 µg/kg), aflatoxin B1 (201.16 µg/kg), zearalenone (1675.46 µg/kg), T-2 toxin (7721.41 µg/kg), and deoxynivalenol (1211.77 µg/kg). Birds were allocated to five groups (*n* = 18/group): CON (basal diet); MYC (MYC diet alone); and TRT1–TRT3 fed the MYC diet supplemented with 1.5 g/kg of MBA I (*Bacillus subtilis* + *Bacillus licheniformis* + *Devosia* sp.), MBA II (*Bacillus licheniformis* + *Saccharomyces cerevisiae*), or MBA III (*Bacillus subtilis* + glucose oxidase), respectively. Subchronic co-exposure to the MYC diet significantly increased serum pro-inflammatory cytokines (TNF-α, IL-1β, IL-6), whereas all three MBAs effectively attenuated these elevations. Histopathological examination revealed inflammatory infiltration and tissue damage in the jejunum and liver of MYC birds, which were markedly alleviated by MBA supplementation. Gut microbiota analysis showed that mycotoxins decreased *Firmicutes* and *Lactobacillus* and increased *Proteobacteria* and *Streptococcus*, whereas MBAs partially restored microbial balance. Among the three MBAs, MBA II exhibited the strongest protective effect, as reflected by the greatest improvements in serum biochemistry, intestinal morphology, and microbial composition. Liver transcriptome analysis showed that mycotoxin mainly affected pathways related to cell cycle and DNA replication, whereas MBA II enhanced pathways associated with intestinal immune function and antigen processing and presentation. Collectively, these findings suggest that MBA II offers effective protection against combined mycotoxin-induced toxicity in pigeons and may serve as a promising feed additive for mitigating mycotoxin contamination in poultry production.

## 1. Introduction

Toxic secondary metabolites known as mycotoxins are generated by several fungal species, mainly from the *Aspergillus*, *Penicillium*, and *Fusarium* genera [1]. The most prevalent mycotoxins in animal feed include aflatoxins (AF), deoxynivalenol (DON), fumonisin (FB),T2 toxin (T2), and zearalenone (ZEN) [2]. In China, surveys conducted between 2018 and 2020 revealed that the incidence of individual mycotoxins in feed samples exceeded 81.9% for AFB1, 96.4% for DON, and 96.9% for ZEN [3]. Mycotoxin contamination not only compromises the nutritional quality of feed but also poses serious health risks, including hepatic and renal damage, immune suppression, and reduced production performance in livestock [4]. For example, feeding a DON-contaminated diet has been shown to exacerbate *Salmonella* dissemination in pigeon flocks through increased bacterial shedding and aggravated paratyphoid lesions [5], while AFB1 exposure can impair growth, alter biochemical indicators, and cause liver and kidney dysfunction in poultry [6,7].

The gastrointestinal tract is the primary target of mycotoxin exposure in both humans and animals [8]. DON disrupts gut barrier function by inducing cellular damage and downregulating tight junction proteins, including occludin and ZO-1 [9], and activates the MAPK signaling pathway, triggering apoptosis and inflammatory responses in intestinal epithelial cells [10]. These effects lead to morphological alterations such as villous atrophy, crypt hyperplasia, and reduced absorptive surface area, ultimately diminishing feed conversion efficiency in poultry [11]. The liver, as the central organ of metabolism, is another primary target of mycotoxin-induced toxicity, with injury mechanisms involving inflammation, oxidative stress, apoptosis, and autophagy [12]. Focal inflammatory infiltrates and apoptotic cells have been observed in liver tissues of pullets exposed to AFB1 [13].

Given the persistent threat posed by mycotoxins, three primary detoxification strategies are currently employed. Chemical/physical adsorbents (e.g., bentonites, zeolites, activated charcoal) act through physical sequestration in the gastrointestinal tract via electrostatic interactions and hydrogen bonds, reducing mycotoxin absorption and hepatic exposure. However, their efficacy is often mycotoxin-specific, pH-dependent, and may be compromised by nutrient binding [14,15]. Biological detoxification agents employ three principal mechanisms: (i) cell wall-mediated binding, in which *S. cerevisiae* β-1,3/1,6-glucans and mannoproteins sequester AFB1, ZEN, and FB1 through non-covalent interactions [16]; (ii) enzymatic degradation, exemplified by *Devosia* mutans de-epoxidizing DON via a cytochrome P450 system, and by carboxylesterases cleaving ZEN and AFB1 [17]; and (iii) gut microbiota modulation, whereby probiotics strengthen the intestinal barrier and reduce inflammation, indirectly alleviating hepatic metabolic stress. At the hepatic level, biological detoxifiers attenuate oxidative stress and pro-inflammatory cytokines through downregulation of NF-κB and upregulation of Nrf2 signaling [18,19,20]. More recently, natural remedies, including plant extracts, essential oils, polyphenols, and traditional herbal compounds, have also been explored as alternative or complementary mycotoxin-detoxifying agents, owing to their antioxidant, antimicrobial, and hepatoprotective properties [21].

Despite these advances, most previous studies have focused on a single adsorbent or a single mycotoxin, and the comparative efficacy of multi-component biological adsorbents against naturally co-occurring mycotoxin cocktails in non-conventional poultry species (such as pigeons) remains poorly understood. This gap holds particular significance as, in actual feeding situations, it is common for various mycotoxins to coexist in tainted feed, and their cumulative toxicity can either be additive or synergistic.

Consequently, this study aimed to assess the protective impacts of three mycotoxin biodegradation agents (MBAs) in a pigeon model, focusing on liver health, intestinal structure, and gut microbiota composition, with liver transcriptome analysis used to clarify the potential molecular mechanisms involved. The results are anticipated to offer a scientific foundation for the formulation of strategies aimed at preventing and controlling mycotoxins in pigeon production.

## 2. Results

### 2.1. Mycotoxin Levels in Mycotoxin-Contaminated Diet (MYC)

The concentrations of main mycotoxins, including FB1, AFB1, ZEN, T-2, and DON, were quantified using high-performance liquid chromatography–tandem mass spectrometry (HPLC-MS/MS). As summarized in Table 1, the basal diet contained low but detectable background levels of FB1, AFB1, ZEN, and DON, while T-2 was below the limit of detection. In the final MYC diet, the concentrations of all five mycotoxins were substantially elevated, reaching 7829.41 ± 1729.47 μg/kg for FB1, 201.16 ± 4.11 μg/kg for AFB1, 1675.46 ± 50.47 μg/kg for ZEN, 7721.41 ± 1067.92 μg/kg for T-2, and 1211.77 ± 358.76 μg/kg for DON. Compared with the GB 13078-2017 regulatory limits, T-2, AFB1, ZEN, and DON exceeded their respective limits by approximately 15.44-, 4.02-, 3.35-, and 1.21-fold, while FB1 remained below its limit.

### 2.2. Clinical Signs

No clinical abnormalities or mortality were observed in the control (CON) group (Table 2). In the MYC treated group, 3/18 pigeons died, and surviving birds developed severe diarrhea (+++), moderate appetite loss, ruffled feathers, and reduced activity (++) from day 10 onwards. These signs were alleviated in all three MBA-treated groups (TRT1, TRT2, and TRT3), with TRT2 (MYC diet + MBA II) showing the strongest improvement (1/18 mortality, mild diarrhea, near-normal activity). TRT1 and TRT3 showed intermediate effects (2/18 and 1/18 mortality, moderate symptoms). These results indicate that combined mycotoxin exposure induced obvious clinical signs in pigeons and that MBAII provided the greatest protection.

### 2.3. Mycotoxin-Induced Inflammation Attenuated by Dietary Adsorbents

Exposure to mycotoxins through diet led to a significant increase in serum levels of tumor necrosis factor-α (TNF-α), interleukin 6 (IL-6), IL-1β, and endothelin (ET) with a statistical significance of *p* < 0.05 (Figure 1). In contrast, a downward trend was noted for the anti-inflammatory cytokine IL-4, where *p* > 0.05 was observed. Supplementation with MBAs effectively alleviated these changes, significantly reducing the serum levels of TNF-α, IL-6, IL-1β, and ET compared with the MYC group (*p* < 0.05). Among the three MBA treatments, the TRT2 group (MYC diet + MBA II) exhibited the most pronounced reductions in these pro-inflammatory markers. Furthermore, IL-4 levels in the TRT2 group were notably greater than those recorded in TRT1 (MYC diet + MBA I, *p* < 0.05) and were comparable to those in TRT3 (MYC diet + MBA III, *p* > 0.05).

### 2.4. Dietary Mycotoxin Adsorbents Effectively Alleviate Mycotoxin-Induced Hepatotoxicity

The activities of serum enzymes recorded at the conclusion of the study are illustrated in Figure 2. When compared to the CON group, the aspartate aminotransaminase (AST) level was significantly increased by mycotoxin exposure (MYC group, *p* < 0.05), while alanine aminotransaminase (ALT) levels did not show a significant variation (*p* > 0.05). The incorporation of MBAs resulted in lower concentrations of both AST and ALT; however, no significant differences were noted (*p* > 0.05).

### 2.5. Dietary Mycotoxin Adsorbents Effectively Mitigate Mycotoxin-Induced Tissue Damage

The histopathological effects of combined mycotoxin exposure on the pigeon liver are shown in Figure 3. The CON group displayed normal hepatic architecture, with radially arranged hepatocyte cords and regular, narrow sinusoidal spaces. In contrast, the MYC group exhibited pronounced lesions, including extensive hepatocyte vacuolar degeneration (steatosis), sinusoidal congestion, focal infiltration of inflammatory cells, disorganization of hepatic cords, and scattered nuclear pyknosis, indicating substantial hepatocellular injury. All three adsorbents alleviated these lesions to varying degrees. TRT2 showed the most pronounced improvement, with hepatocyte morphology and tissue architecture approaching those of the CON group, indicating the strongest hepatoprotective effect. TRT1 and TRT3 also showed clear alleviation, with reduced vacuolation and inflammatory infiltration, although mild residual damage remained. These findings confirm that combined mycotoxin exposure induced severe hepatocellular injury in pigeons and that all three adsorbents, particularly MBA II, effectively attenuated these alterations, supporting the serum biochemical results.

As shown in Figure 4, the jejunal microstructures presented the marked difference among the different treatment groups. The CON group displayed normal architecture with tall villi, intact epithelium, and organized crypts. Conversely, the MYC group demonstrated significant mucosal injury, including villous atrophy and fusion, loss of epithelial cells, dense inflammatory infiltration, crypt disorganization, and submucosal edema. The TRT2 and TRT3 groups demonstrated a notable recovery, with villus height comparable to that of the control and tightly interconnected epithelial cells, indicating well-preserved mucosal morphology.

### 2.6. Mycotoxin Adsorbent Restores Gut Microbial Unbalance Under Mycotoxin-Induced Stress

To assess the impact of mycotoxin exposure and MBA supplementation on the gut bacterial community, 16S rRNA sequencing was performed on jejunal contents obtained from the five pigeon groups. As illustrated in Figure 5a, the Chao1, Simpson, and Shannon indices showed no significant differences across the groups (*p* > 0.05), indicating that neither the challenge with mycotoxins nor the MBA supplementation had a substantial effect on the α-diversity of the gut microbiota. In contrast, PCA revealed a clear separation in microbial community structure, suggesting notable changes in β-diversity (Figure 5b).

Analysis of microbial composition further demonstrated notable variations at both the phylum and genus levels among the various treatments (Figure 5c,d). At the phylum level, *Firmicutes*, *Actinobacteriota*, *Proteobacteria*, and *Cyanobacteria* were the prevailing group in the microbiota of the control group. Mycotoxin exposure reduced the abundance of *Firmicutes* and increased that of *Proteobacteria*, while MBA supplementation effectively reversed these changes (Figure 5c). At the genus level, the five most abundant taxa were *Lactobacillus*, *Corynebacterium*, *Bacillus*, *Enterococcus*, and *Veillonella* (Figure 5d). Compared to other groups, the MYC group showed significantly lower abundances of *Lactobacillus* and *Bacillus*, and along with a higher abundance of *Enterococcus* (*p* < 0.05; Figure 5e,f). Among the three MBA-treated groups, TRT2 exhibited the highest abundance of *Lactobacillus* and the lowest abundance of *Enterococcus*. Collectively, these findings indicated that the MBAs, particularly in the TRT2, were contributed to restore a more favorable gut microbial composition under mycotoxin-induced stress.

### 2.7. Mycotoxin Adsorbent Modulate Hepatic Transcriptomic Profiles After Mycotoxin Exposure

Based on previous protective effects of TRT2 across multiple parameters including serum inflammatory markers, enzyme activities, histopathology, and gut microbiota, we performed transcriptomic sequencing to elucidate the underlying hepatic protective mechanism of MBA II. RNA-seq was conducted on liver samples from three groups: the CON group (basal diet), the MYC group (MYC diet alone), and the TRT2 group (MYC diet + MBA II).

Principal component analysis (PCA) demonstrated clear clustering of hepatic transcriptomic profiles (Figure 6b). Samples from the CON group clustered tightly, indicating low intra-group variability. In contrast, the MYC group was markedly distinct from the CON group, reflecting substantial mycotoxin-induced alterations in gene expression. The TRT2 group exhibited an intermediate profile that lay between the MYC and CON groups, suggesting that the MBA partially reversed the mycotoxin-driven transcriptional changes.

Differentially expressed gene (DEG) analysis identified 442 DEGs a total of 442 DEGs in the MYC group when contrasted with the control, comprising 374 that were upregulated and 68 that were downregulated (Figure 6a). In the TRT2 group, 549 DEGs (471 upregulated, 78 downregulated) were detected compared to the control group. In addition, comparison between the TRT2 and MYC groups, there was 181 DEGs identified with 81 upregulated and 100 downregulated, indicating a meaningful transcriptional response to adsorbent intervention.

Gene Ontology (GO) enrichment analysis of the DEGs identified 20 significantly enriched GO terms across the three major functional categories (Figure 6c). In the biological process domain, DEGs were primarily involved in signal transduction, such as cellular metabolic processes, and regulation of biological processes. Within the cellular component category, genes were mainly enriched in membrane-related structures, organelles, and intrinsic components of the membrane. For molecular function, key terms included protein binding, transferase activity, and transmembrane transporter activity.

Kyoto Encyclopedia of Genes and Genomes (KEGG) pathway analysis further elucidated the metabolic and signaling pathways modulated by mycotoxin exposure and adsorbent intervention (Figure 6d,e). The top 10 pathways enriched in CON vs. MYC comparison were included cell cycle, amoebiasis, DNA replication, arginine biosynthesis, malaria, cytokine–cytokine receptor interaction, ABC transporters, progesterone-mediated oocyte maturation, calcium signaling pathway, and Staphylococcus aureus infection. In the MYC vs. TRT2 comparison (Figure 6e), the most significantly enriched pathways were Staphylococcus aureus infection, intestinal immune network for IgA production, asthma, systemic lupus erythematosus, allograft rejection, inflammatory bowel disease, autoimmune thyroid disease, antigen processing and presentation, nitrogen metabolism, and graft-versus-host disease.

## 3. Discussion

The combined mycotoxin concentrations in the MYC diet exceeded the regulatory limits specified in GB 13078-2017 for four of the five target toxins, with T-2, AFB1, ZEN, and DON exceeding their respective limits by 15.44-, 4.02-, 3.35-, and 1.21-fold (Table 1). These concentrations are within the upper range of mycotoxin levels reported in field surveys of naturally contaminated poultry feed, indicating that the present model represents a moderately-to-severely contaminated feed scenario that is environmentally relevant for commercial pigeon production. The naturally mold-contaminated feed model used here has been widely employed to evaluate mycotoxin toxicity and the efficacy of detoxifying agents in various poultry species [22,23].

Consistent with the observed histopathological damage, mycotoxin exposure significantly increased serum pro-inflammatory cytokines and decreased the anti-inflammatory cytokine IL-4. These effects are likely attributable to the well-documented activation of the NF-κB signaling pathway by DON, and AFB1, which induces the transcription of pro-inflammatory cytokine genes [24,25]. All three MBAs attenuated these inflammatory responses, with MBA II showing the most pronounced effect. The enhanced effectiveness of MBA II is probably due to its ingredients: β-glucans and mannoproteins from the cell wall of *S. cerevisiae* have the ability to bind mycotoxins within the gastrointestinal system, creating stable complexes that are eliminated through feces [26], while *B. licheniformis* has been shown to enhance innate immunity and modulate cytokine responses in poultry [27,28]. Together, these components may provide complementary anti-inflammatory effects, although the precise contributions of each mechanism remain to be elucidated.

Under normal physiological conditions, the activities of AST and ALT in the serum stayed low; however, hepatocellular injury caused these enzymes to be released into the bloodstream, leading to an increase in their serum concentrations [29]. The elevated activities of AST and ALT in serum, which indicate hepatocellular damage, alongside the histopathological findings in the liver, clearly demonstrated the hepatotoxic effects of mycotoxins. Pathological alterations in the livers of geese were noted, which encompassed disrupted hepatic lobular structure, chaotic arrangement of hepatic cords, slight hydropic degeneration of hepatocytes, and infiltration of perivascular mononuclear cells as a reaction to exposure to low levels of AFB1 (10 μg/kg) [30]. The addition of a triple-action mycotoxin detoxifier, composed of AFB1-degrading enzyme, montmorillonite, and a composite probiotic blend, has been shown to alleviate mycotoxin-induced liver tissue damage of broilers [31].Consistent with previous studies [22,30], the inclusion of MBAs reduced serum AST and ALT activities, although the levels did not fully return to those of the CON group, suggesting that the 60-day exposure produced substantial hepatocellular damage that could not be completely reversed by dietary intervention alone.

Furthermore, mycotoxin exposure caused marked jejunal damage, including villus atrophy and fusion, epithelial desquamation, and inflammatory cell infiltration, consistent with the well-documented intestinal toxicity of DON, AFB1, and T-2 in poultry [30,31]. The villus-crypt architecture is critical for nutrient absorption and barrier function, and its disruption directly contributes to the reduced feed conversion efficiency commonly observed in mycotoxin-exposed birds [31]. MBA II again exhibited the strongest protective effect, which may be attributed to the combined actions of *S. cerevisiae* cell wall β-glucans (mycotoxin sequestration) and *B. licheniformis* (immune modulation and competitive exclusion of pathogens) [32,33]. Notably, the close interplay between intestinal structural integrity and microbial homeostasis prompted us to further investigate the gut microbiota.

The gut microbiota plays a crucial role in preserving the health of the host, while microbial dysbiosis is often associated with a range of functional disorders. In ecological terms, α-diversity reflected the species richness and evenness with a community, whereas β-diversity captured the structural differences across samples or individuals [34]. In the present study, mycotoxin exposure did not significantly alter α-diversity but induced clear shifts in β-diversity, indicating compositional rather than richness-based dysbiosis. At the genus level, mycotoxin exposure reduced abundance of beneficial bacteria (*Lactobacillus*, *Bacillus*, *Veillonella*) while promoting potentially pathogenic taxa (*Enterococcus*), a pattern consistent with previous studies in mice and laying hens exposed to ZEN or AFB1 [35,36,37]. MBA II most effectively counteracted these alterations, which may be explained by the documented ability of both *B. licheniformis* and *S. cerevisiae* to promote the growth of *Lactobacillus* through cross-feeding and short-chain fatty acid production [38,39,40]. *Lactobacillus* species, in turn, enhance mucus barrier production and secrete antimicrobial peptides that suppress pathogen colonization [36], forming a positive feedback loop that contributes to microbial homeostasis. These findings might suggest that the probiotic components of the MBAs play an important role in mitigating mycotoxin-induced dysbiosis, in addition to their direct mycotoxin-binding activities.

To further elucidate the molecular basis of mycotoxin-induced hepatotoxicity and MBA II-mediated protection, liver transcriptomic analysis was performed on the CON, MYC, and TRT2 groups. Consistent with the observed hepatic histopathology, KEGG pathway analysis revealed that DEGs in the MYC group showed significant enrichment in pathways related to the cell cycle and DNA replication. This finding suggests that mycotoxins negatively affect the proliferation of hepatocytes and the synthesis of DNA. This observation aligns with the documented capacity of AFB1 to increase the levels of p21/p53 and trigger G1/S or G2/M cell cycle arrest [41,42], and of DON to activate p38 MAPK/JNK signaling, leading to CDK1/Cyclin B1 suppression and G2/M arrest [43,44]. These alterations led to impaired cellular function and accumulation of necrotic cells, thereby triggered a robust inflammatory response [45]. In parallel, the enrichment of DNA replication pathways implies that mycotoxins may directly interfere with DNA synthesis and repair, as supported by reports that OTA exposure elevates γ-H2AX and DNA repair-related gene expression, and that replication stress can trigger cGAS-STING-mediated hepatic inflammation [46,47].

Notably, DEGs in the TRT2 group exhibited a notable enrichment in the pathways of “antigen processing and presentation” as well as “intestinal immune network for IgA production” when compared to the MYC group. This finding suggests that MBA II may alleviate mycotoxin-induced hepatic inflammation partly by enhancing mucosal IgA production and antigen presentation, both of which are fundamental to adaptive immunity. Components of the cell wall in *S. cerevisiae*, such as β-glucans and mannan oligosaccharides, along with short-chain fatty acids generated by *B. licheniformis*, are recognized for their ability to stimulate the differentiation of B cells within the mucosal tissue into plasma cells that secrete IgA. Additionally, they enhance the presentation of antigens through MHC class I and II molecules to CD8+ and CD4+ T lymphocytes, respectively [48,49]. Although the cross-organ mechanism linking intestinal immune enhancement to hepatic protection remains to be fully elucidated, these transcriptomic findings provide a plausible molecular basis for the observed protective effects of MBA II.

The presence of several mycotoxins in feed is common rather than unusual, and exposure to these combinations can lead to either synergistic or additive toxic effects—especially in cases where AFB1 is combined with FB1, ZEN, or T-2 [23,26]. The current MYC diet featuring a five-mycotoxin cocktail illustrates a practical scenario of multi-mycotoxin exposure, which is well-suited for protection by the multi-component MBAs. The enhanced effectiveness of MBA II probably results from the complementary mechanisms of *B. licheniformis* (which offers binding and enzymatic activity) alongside *S. cerevisiae* (known for β-glucan-mediated adsorption of its cell wall), allowing for the simultaneous targeting of various toxins. From a public health standpoint, effectively mitigating multi-mycotoxin contamination in animal feed may also diminish the carryover of mycotoxins into food products derived from animals, consequently reducing human dietary exposure.

Several limitations of the current research must be recognized. To begin with, the transcriptomic results were not validated independently through targeted qPCR or functional assays and should thus be considered exploratory and hypothesis-generating instead of definitive mechanistic conclusions. Additionally, the lack of MBA-only control groups (i.e., MBA given in a mycotoxin-free diet) prevents a complete differentiation between mycotoxin sequestration and the inherent bioactive properties of the MBA components. Furthermore, the feed naturally contaminated with mold contained a complex and variable array of mycotoxins, complicating the attribution of the observed effects to any individual toxin. Moreover, clinical signs were evaluated descriptively instead of through a standardized scoring system, which may have introduced some level of subjectivity. Lastly, the artificially mold-contaminated feed was produced under controlled laboratory conditions, which cannot entirely replicate the intricacies of natural mold proliferation found in real-world feed storage settings. Future research that includes transcriptomic validation, MBA-only controls, purified mycotoxin standards, single-strain fungal cultures, and standardized clinical assessments is advisable.

## 4. Conclusions

In summary, subchronic co-exposure to a diet contaminated with multiple mycotoxins induced significant adverse effects in pigeons. The combined exposure to FB1, AFB1, ZEN, T-2, and DON toxins resulted in: (i) diarrhea and a mortality rate of 3 out of 18 pigeons; (ii) significant elevations in serum TNF-α, IL-1β, IL-6, ET, and AST; (iii) histopathological damage in the jejunum and liver; and (iv) dysbiosis of gut microbiota, characterized by decreased levels of *Firmicutes* and *Lactobacillus* and increased levels of *Proteobacteria* and *Streptococcus*. Among the three MBAs, MBA II, consisting of *B. licheniformis* and *S. cerevisiae*, consistently exhibited the strongest protective effects across all measured endpoints. This efficacy is likely attributed to its multi-component composition, which provides complementary mechanisms for mycotoxin sequestration and probiotic modulation. Liver transcriptome analysis indicated that mycotoxin exposure primarily affected pathways related to the cell cycle and DNA replication, whereas MBA II enhanced pathways associated with intestinal immune function and antigen processing. Collectively, MBA II may serve as a promising multi-component feed additive for mitigating combined mycotoxin contamination in pigeon and poultry production.

## 5. Materials and Methods

### 5.1. Preparation of Mycotoxin-Contaminated Diet

Commercially available pigeon pelleted feed (Shanghai Hongma Feed Co., Ltd., Shanghai, China) was used as the basal feed for mold contamination. The feed was spread in a layer approximately 2 cm thick in a closed, sterile chamber and incubated under strictly controlled environmental conditions (25 ± 2 °C, 85 ± 5% relative humidity) to allow spontaneous colonization by ambient fungi, without artificial inoculation with specific fungal spores. All batches were incubated until reaching a uniform visual endpoint defined as >80% surface coverage by visible fungal colonization, ensuring consistent colonization intensity across replicates. Three independently prepared batches were produced in parallel, pooled, and thoroughly homogenized to minimize inter-batch variability. The naturally mycotoxin -contaminated diet (MYC) was then prepared by mixing the contaminated pelleted feed at 3% (*w*/*w*) into the standard pigeon basal diet. The concentrations of the major mycotoxins (FB1, AFB1, ZEN, T-2, and DON) in the basal diet and MYC diet were quantified by HPLC-MS/MS.

Chromatographic separation of the targeted mycotoxins was carried out on an Agilent Poroshell 120 EC-C18 column (100 mm × 3.0 mm, 2.5 μm). Briefly, acetonitrile and water containing 0.1% formic acid (for FB1) or 5 mmol/L ammonium acetate (for other mycotoxins) were used as the mobile phases under a gradient elution. The flow rate was 0.4 mL/min, injection volume 3 μL, and column temperature 40 °C. Mass spectrometric detection was performed in both positive and negative electrospray ionization modes under multiple reaction monitoring. Other mass spectrometry parameters are detailed in Table 3.

### 5.2. Experimental Birds, Design, and Management

A total of 90 pigeons of similar weight (566.78 ± 26.47 g) at 30 days of age were randomly assigned to five treatment groups (*n* = 18/group), with equal numbers of males. Each group comprised six replicates, with three pigeons per replicate in one cage. The recommended rearing temperature is 15–25 °C, with humidity levels between 45% and 55%. The duration of experiment was 67 days, including a 7-day adaptation period. The composition and nutrient levels of the basal diet are presented in Table 4. Feed and water were provided *ad libitum* throughout the study.

Five experiment groups were set up as follows:

CON (control): fed the standard pigeon basal diet.

MYC: fed a diet containing 97% (*w*/*w*) standard pigeon basal diet plus 3% (*w*/*w*) naturally mold-contaminated pelleted feed.

TRT1: fed the MYC diet supplemented with MBA I, which contained *Bacillus subtilis* ANSB060 (6 × 10^8^ CFU/g), *Bacillus licheniformis* (5 × 10^8^ CFU/g), and *Devosia* sp. ANSB714 (7.5 × 10^8^ CFU/g).

TRT2: fed the MYC diet supplemented with MBA II, which contained *Bacillus licheniformis* (5 × 10^8^ CFU/g) and *Saccharomyces cerevisiae* EMCC 97 (7.5 × 10^8^ CFU/g).

TRT3: fed the MYC diet supplemented with MBA III, which contained *Bacillus subtilis ANSB060* (6 × 10^8^ CFU/g) and glucose oxidase (10,000 U/g; Sigma-Aldrich, St. Louis, MO, USA).

All bacterial strains were obtained from the BeNa Culture Collection (Hebei, China). Each MBA was supplemented at a dose of 1.5 g/kg of the MYC diet, thoroughly mixed prior to feeding.

Throughout the experimental period, pigeons were fed a fixed daily ration of 50 g per bird, and the amount of feed offered was adjusted according to the actual feed intake of each pigeon to ensure complete consumption of the supplied diet within 24 h. The contaminated pelleted feed was prepared in a single batch, portioned into daily aliquots according to individual feed intake, and stored at −20 °C in airtight containers until use. For each feeding, the required amount of contaminated pelleted feed was freshly thawed and thoroughly mixed with the corresponding amount of basal feed immediately prior to administration. This freshly prepared, on-demand mixing procedure was adopted to maintain the stability and consistency of the mycotoxin challenge materials across the entire experimental period.

### 5.3. Clinical Observations

Throughout the 60-day experimental period, all pigeons were monitored twice daily (morning and afternoon) for clinical signs, including general behavior, appetite, fecal consistency and color, and mortality. Any abnormal clinical manifestations (e.g., lethargy, ruffled feathers, diarrhea, emaciation) were recorded descriptively. Dead birds, if any, were necropsied to determine the cause of death.

### 5.4. Sample Collection

After excluding mortalities throughout the 67-day trial, after a 12 h fasting period, approximately 2.5–3.0 mL of blood was drawn from the jugular vein of each pigeon (*n* = 15/group) for serum biochemical analysis. Samples were incubated at 4 °C for 2 h, and serum was separated by centrifugation at 1500× *g* for 10 min, then stored at −20 °C. At the end of the 67-day experimental period, all pigeons were humanely euthanized by cervical dislocation after overnight fasting. For tissue collection, eight pigeons per group (*n* = 8/group) were randomly selected, euthanized, and ileal content was collected, and then the liver and ileum tissues were dissected and surgically removed. All samples were promptly frozen in liquid nitrogen. In addition, fresh samples of liver and ileum (0.5 cm^3^) were preserved in a 10% formalin solution (pH 7.4) and kept at 4 °C for histopathological study.

### 5.5. Serum Biochemical Parameters

To examine liver damage caused by exposure to mycotoxins, two biochemical markers, namely AST and ALT, were analyzed. Additionally, serum levels of inflammatory factors such as TNF-α, IL-4, IL-6, IL-1β, and ET were quantified using commercial kits from the Nanjing Jiancheng Institute of Biological Engineering (Nanjing, China). Following the manufacturer’s instructions, all biochemical markers and inflammatory factors were evaluated with a microplate reader and a standard curve.

### 5.6. Histopathological Analysis

After 24 h of fixation, liver and jejunum samples were moved to 70% ethanol and then dehydrated using a sequence of alcohol solutions (80%, 90%, 100%). After xylene clearing, tissues were embedded in paraffin wax. To stain the slices, they were dewaxed in xylene, rehydrated using 95% and 80% ethanol, and rinsed with distilled water. Hematoxylin and eosin staining was then conducted according to standard protocols. Sections were dehydrated with increasing ethanol concentrations (80%, 95%, 100%) and xylene. Ultimately, the Panoramic Desk Histological Blade Scanner from 3D Histech in Hungary was employed to scan the slides and obtain images using the panoramic viewer software from the same company.

### 5.7. Intestinal Microbiota Analysis

Ileal content samples from each group were used for microbiome profiling analysis. Total genomic DNA was extracted using the FastPure Stool DNA Isolation Kit (MJYH, Shanghai, China). DNA quality and concentration were evaluated using 1.0% agarose gel electrophoresis and a NanoDrop2000 spectrophotometer (Thermo Scientific, Waltham, MA, USA).

The hypervariable V3-V4 region of the bacterial 16S rRNA gene were amplified with primer pairs 338F (5′-ACTCCTACGGGAGGCAGCAG-3′) and 806R (5′-GGACTACHVGGGTWTCTAAT-3′) using a T100 Thermal Cycler PCR thermal Cycler (Bio-Rad, Minneapolis, MN, USA). PCR products were purified using the PCR Clean-Up Kit (YuHua, Shanghai, China), and quantified using a Qubit 4.0 fluorometer (Thermo Fisher Scientific, Waltham, MA, USA). Purified amplicons in equal molar quantities were combined and sequenced using paired-end technology on an Illumina NextSeq2000 platform (Illumina, CA, USA) by Majorbio Bio-Pharm Technology Co., Ltd. (Shanghai, China), adhering to standard procedures.

Bioinformatic analysis was conducted on the Majorbio Cloud platform (https://cloud.majorbio.com). Based on the amplicon sequence variant data, rarefaction curves and alpha diversity indices (Ace, Chao1, Shannon, and Sobs) were calculated using Mothur v1.30.1. Beta diversity was evaluated through principal coordinate analysis (PCoA) based on Bray–Curtis dissimilarity, employing the Vegan v2.5-3 package. The linear discriminant analysis (LDA) effect size was used to identify differentially abundant bacterial taxa (from phylum to genus) across groups (LDA score > 2, *p* < 0.05).

### 5.8. RNA Sequencing

Total RNA was extracted from pigeon liver tissue using Trizol reagent (Takara, Dalian, China) with genomic DNA removed during extraction. The RNA integrity number was further determined with an Agilent 2100 Bioanalyzer (Agilent Technologies Inc., Santa Clara, CA, USA). RNA library construction and sequencing were performed by Shanghai Majorbio Bio-pharm Biotechnology Co., Ltd. (Shanghai, China). In brief, transcriptome libraries were constructed from 1 μg of total RNA using the Illumina TruSeq RNA Sample Preparation Kit (Illumina, San Diego, CA, USA). Gene expression levels were quantified as fragments per kilobase of transcript per million mapped reads to DEGs between groups. Gene abundance estimation was conducted using RSEM (http://deweylab.github.io/RSEM/ accessed on 8 November 2024). Functional enrichment analysis was carried out based on the GO (http://www.geneontology.org) and KEGG (http://www.genome.jp/kegg/, accessed on 5 November 2024) databases.

### 5.9. Statistical Analysis

GraphPad Prism version 8.00 (La Jolla, CA, USA) was used for all statistical analyses of the experiments, and the results were expressed as the mean ± standard error. A one-way ANOVA followed by a Tukey post hoc test was used to identify significant differences between groups, with significance set at *p* < 0.05.

## Figures and Tables

**Figure 1:**
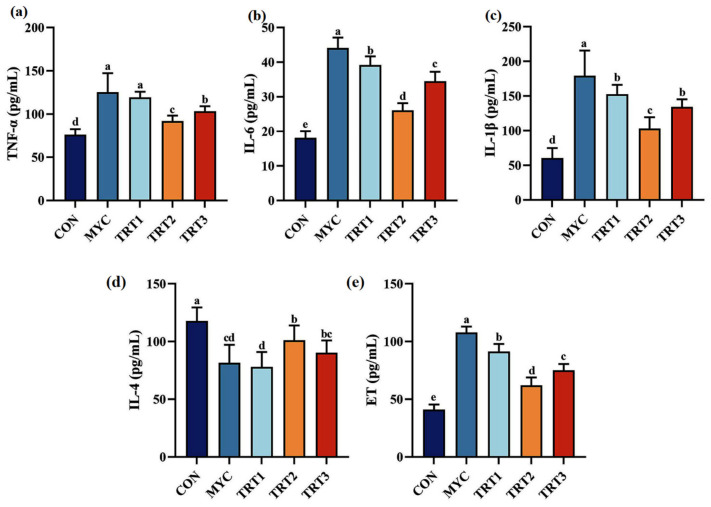
Effects of dietary mycotoxins and mycotoxin biodegradation agents on serum immune parameters in pigeons (*n* = 15). (**a**) Tumor necrosis factor-α (TNF-α); (**b**) Interleukin6 (IL-6); (**c**) Interleukin-1β (IL-1β); (**d**) Interleukin4 (IL-4); (**e**) endothelin (ET); values are presented as mean ± standard error. Bars that lack a shared superscript letter indicate significant differences (*p* < 0.05) as assessed by one-way ANOVA. CON: control group fed the basal diet; MYC: mycotoxin-treated group fed the contaminated diet alone; TRT1, TR2 and TRT3 means the pigeons fed the MYC diet supplemented with mycotoxin biodegradation agent (MBA) I, II, or III at 1.5 g/kg of feed, respectively.

**Figure 2:**
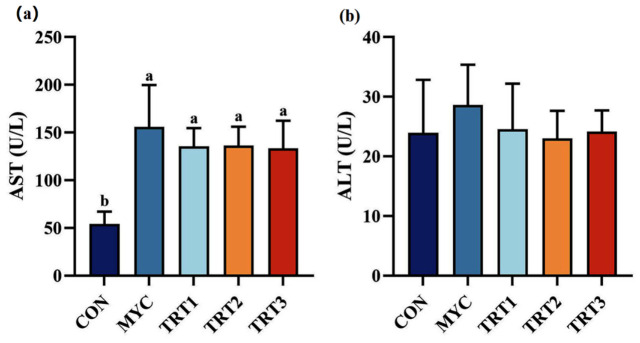
Effects of dietary mycotoxins and mycotoxin adsorbent agents on serum enzyme activities in pigeons (*n* = 15). (**a**) Aspartate aminotransferase (AST). (**b**) Alanine aminotransferase (ALT). CON: control group fed the basal diet; MYC: mycotoxin-treated group fed the contaminated diet alone; TRT1, TR2 and TRT3 means the pigeons fed the contaminated diet supplemented with mycotoxin biodegradation agent (MBA) I, II, or III at 1.5 g/kg of feed, respectively. Values with different lowercase letters (a, b) differ significantly (*p* < 0.05).

**Figure 3:**
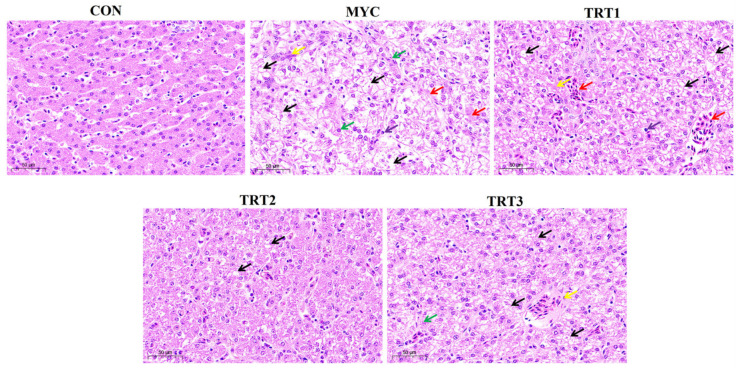
Representative histopathological sections of pigeon liver (H&E, ×100, *n* = 8). Normal hepatic architecture with radially arranged hepatocyte cords and regular sinusoidal spaces in the CON group. Pronounced lesions including hepatocyte vacuolar degeneration (steatosis, black arrowhead), sinusoidal congestion (red arrow), focal inflammatory cell infiltration (yellow arrow), disorganized hepatic cords (green arrow), and scattered nuclear pyknosis (purple arrow) in the MYC group. Moderate alleviation of MYC-induced lesions, with reduced vacuolation and inflammation but mild residual cord disarray in the TRT1 group. The strongest hepatoprotective effect, with minimal vacuolation observed in the TRT2 group. For TRT3 group, clear improvement over MYC, but slightly more residual damage than TRT2 were observed. CON: control group fed the basal diet; MYC: mycotoxin-treated group fed the mycotoxin-contaminated diet alone; TRT1, TR2 and TRT3 means the pigeons fed the mycotoxins contaminated diet supplemented with mycotoxin biodegradation agent (MBA) I, II, or III at 1.5 g/kg of feed, respectively.

**Figure 4:**
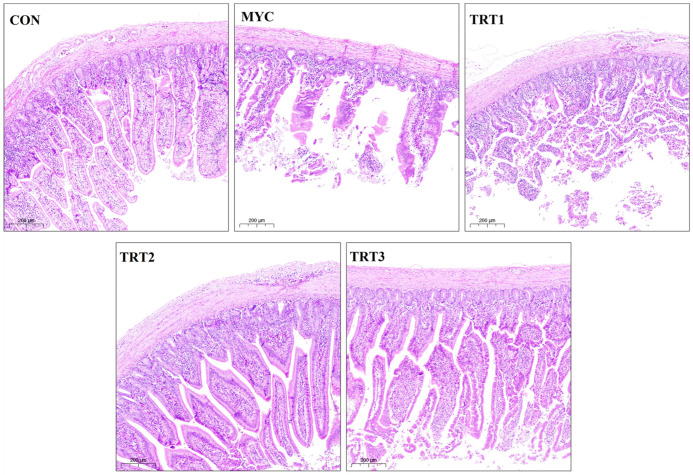
Representative histopathological sections of pigeon jejunum (H&E staining, 100× magnification, *n* = 8). CON: control group fed the basal diet; MYC: mycotoxin-treated group fed the mycotoxin-contaminated diet alone; TRT1, TRT2 and TRT3 means the pigeons fed the mycotoxins contaminated diet supplemented with mycotoxin biodegradation agent (MBA) I, II, or III at 1.5 g/kg of feed, respectively.

**Figure 5:**
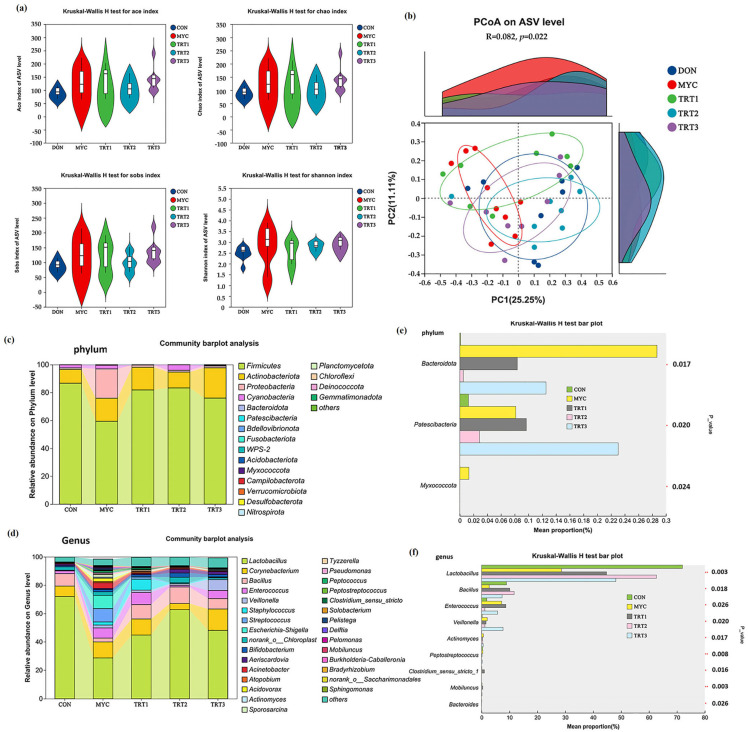
(**a**) Alpha diversity indices. (**b**) Principal coordinate analysis (PCoA). (**c**,**d**) Overall microbial composition at the phylum and genus levels, respectively. (**e**,**f**) Relative abundances of significantly altered microbial taxa at the phylum and genus levels, respectively (*n* = 8). CON: control group fed the basal diet; MYC: mycotoxin-treated group fed the mycotoxin-contaminated diet alone; TRT1, TRT2 and TRT3 means the pigeons fed the mycotoxins contaminated diet supplemented with mycotoxin biodegradation agent (MBA) I, II, or III at 1.5 g/kg of feed, respectively.

**Figure 6:**
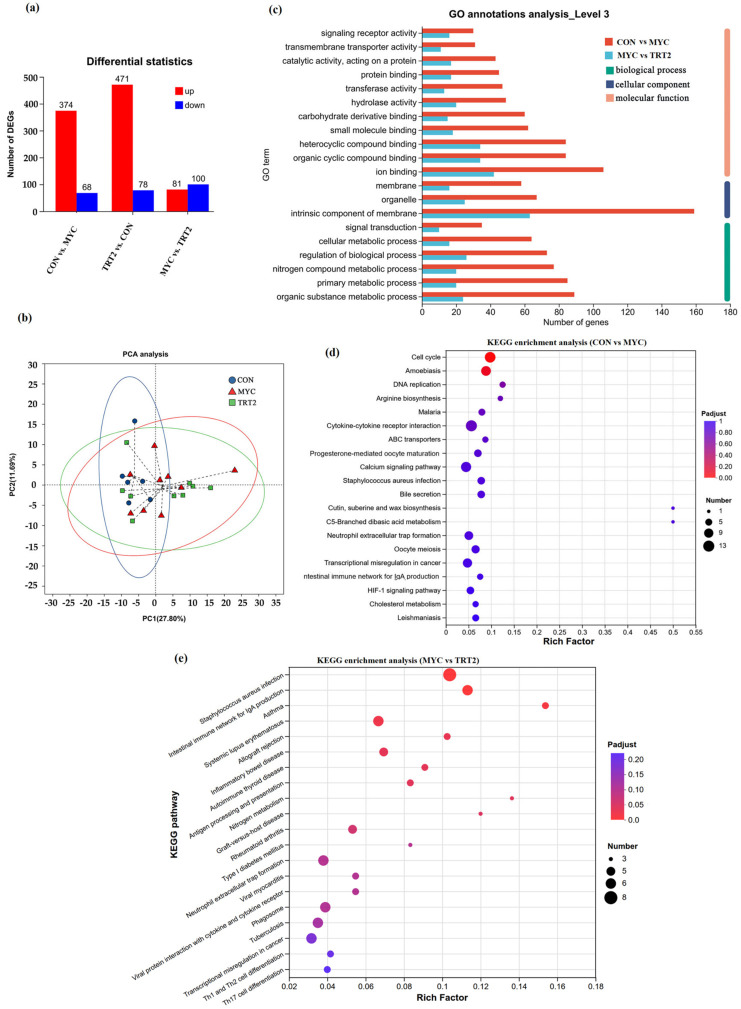
Transcriptomic analysis of liver tissue. (**a**) Differentially expressed genes (DEGs) between CON and MYC groups, CON and TRT2 groups, and MYC and TRT2 groups, respectively. (**b**) Principal component analyses of relationships among samples. (**c**) GO enrichment analysis of differentially expressed genes. (**d**) KEGG enrichment analysis of DEGs in MYC vs. CON group. (**e**) KEGG enrichment analysis of differentially expressed genes in MYC vs. TRT2 group. *n* = 8. CON: control group fed the basal diet; MYC: mycotoxin-challenged group fed the mycotoxin-contaminated diet alone; TRT2: the pigeons fed the mycotoxin-contaminated diet supplemented with MBA II at 1.5 g/kg of feed.

**Table 1:** Mycotoxin concentrations (μg/kg) in the basal diet and the final MYC diet.

Mycotoxins	Basal Diet	MYC	Limited in Feed ^1^	Fold of Limit ^2^
FB1	222.63 ± 68.58	7829.41 ± 1729.47	20,000	0.39
AFB1	4.61 ± 1.58	201.16 ± 4.11	50	4.02
ZEN	40.78 ± 4.89	1675.46 ± 50.47	500	3.35
T-2	-	7721.41 ± 1067.92	500	15.44
DON	180.90 ± 37.43	1211.77 ± 358.76	1000	1.21

^1^ The threshold of Hygienical Standard for Feeds of China (GB 13078–2017). ^2^ Fold of GB 13078-2017 limit. FB1: fumonisin B1; AFB1: aflatoxin B1; ZEN: zearalenone; T-2: trichothecenes; DON: deoxynivalenol. MYC: Mycotoxin-contaminated diet prepared by mixing 3% (*w*/*w*) mycotoxin-contaminated pelleted feed with 97% (*w*/*w*) basal feed. - means undetected.

**Table 2:** Summary of clinical observations in pigeons during the 60-day experimental period.

Group	Mortality	Diarrhea	Appetite Loss	Ruffled Feathers	Reduced Activity
CON	0/18	−	−	−	−
MYC	3/18	+++	++	++	++
TRT1	2/18	++	+	+	+
TRT2	1/18	+	+	+	−/+
TRT3	1/18	++	+	+	+

−: absent; +: mild; ++: moderate; +++: severe. −/+: some animals showed no clinical signs while others displayed mild signs. *n* = 18 per group. CON: control group fed the basal diet; MYC: mycotoxin-treated group fed the contaminated diet alone; TRT1, TR2 and TRT3 means the pigeons fed the contaminated diet supplemented with mycotoxin-detoxifying agents (MDA) I, II, or III at 1.5 g/kg of feed, respectively.

**Table 3:** Mass spectral parameters of 5 mycotoxins.

Mycotoxin	Retention Period (min)	Parent	Daughter	Impulse Voltage (eV)	Ion Source	LOD (μg/kg)	Recovery Rates (%)
FB1	2.97	722.39	334.38 */352.38	38/34	ES+	0.2	98.3
AFB1	3.32	313.24	241.16 */284.97	36/22	ES+	0.05	101.2
ZEN	4.19	317.24	175.05 */131.03	26/24	ES-	0.3	97.6
T-2	4.71	484.1	213.9 */304.9	28/19	ES+	1	98.5
DON	1.99	297.1	249.10 */231.10	13/10	ES+	4	100.2

* Quantitative ion; FB1: fumonisin B1; AFB1: aflatoxin B1; ZEN: zearalenone; T-2: trichothecenes; DON: deoxynivalenol.

**Table 4:** Composition and nutrition level of basal diet (air dry basis).

Ingredients	Content (%)	Nutrient Level ^1^	Content (%)
Raw Grain		ME (MJ/kg)	12.08
Corn	26.4	Crude protein	15.41
Soybean	19.8	Calcium	2.42
Wheat	16.5	Lysine	0.73
Sorghum	3.30	Methionine	0.41
Pelleted feed			
Corn	10.88		
Soybean	4.08		
Wheat	1.02		
Soybean meal	11.22		
Wheat skin	0.51		
Wheat middlings	1.70		
Conch meal	0.34		
Limestone	0.34		
CaHPO4	0.34		
Soya-bean oil	3.40		
Nacl	0.07		
Premix ^2^	0.10		
Total	100		

^1^ The nutrient content was measured by the mixture of raw grain and pelleted feed except the ME. The experimental diets were formulated to contain similar levels of metabolic energy (ME) and crude protein (CP) to meet the nutritional needs of pigeons according to the NRC (Nutrient requirements of poultry. Washington, DC: The National Academies Press, 1994). ^2^ The premix provided the follows per kilogram of diet VA 3200 KIU, VD3 800 KIU, VE ISIU, VK 16 mg, VBl 8 mg, VB2 24.8 mg, Calcium pathtothenate 400 mg, niacin 32.5 mg, VB6 64 mg. VB12 5 mg, biotin 2 mg, Fe 60 mg, Zn 66 mg, Mn 65 mg, Se 0.3 mg, I l mg.

## Data Availability

The original contributions presented in the study are included in the article, further inquiries can be directed to the corresponding authors.
